# Classifying kinase conformations using a machine learning approach

**DOI:** 10.1186/s12859-017-1506-2

**Published:** 2017-02-02

**Authors:** Daniel Ian McSkimming, Khaled Rasheed, Natarajan Kannan

**Affiliations:** 1 Institute of Bioinformatics, University of Georgia, Athens, GA 30602 USA; 2 Department of Computer Science, University of Georgia, Athens, GA 30602 USA; 3 Department of Biochemistry & Molecular Biology, University of Georgia, Athens, GA 30602 USA

**Keywords:** Kinase conformation, Machine learning, Classifier, Activation segment

## Abstract

**Background:**

Signaling proteins such as protein kinases adopt a diverse array of conformations to respond to regulatory signals in signaling pathways. Perhaps the most fundamental conformational change of a kinase is the transition between active and inactive states, and defining the conformational features associated with kinase activation is critical for selectively targeting abnormally regulated kinases in diseases. While manual examination of crystal structures have led to the identification of key structural features associated with kinase activation, the large number of kinase crystal structures (~3,500) and extensive conformational diversity displayed by the protein kinase superfamily poses unique challenges in fully defining the conformational features associated with kinase activation. Although some computational approaches have been proposed, they are typically based on a small subset of crystal structures using measurements biased towards the active site geometry.

**Results:**

We utilize an unbiased informatics based machine learning approach to classify all eukaryotic protein kinase conformations deposited in the PDB. We show that the orientation of the activation segment, measured by φ, ψ, χ1, and pseudo-dihedral angles more accurately classify kinase crystal conformations than existing methods. We show that the formation of the K-E salt bridge is statistically dependent upon the activation segment orientation and identify evolutionary differences between the activation segment conformation of tyrosine and serine/threonine kinases. We provide evidence that our method can identify conformational changes associated with the binding of allosteric regulatory proteins, and show that the greatest variation in inactive structures comes from kinase group and family specific side chain orientations.

**Conclusion:**

We have provided the first comprehensive machine learning based classification of protein kinase active/inactive conformations, taking into account more structures and measurements than any previous classification effort. Further, our unbiased classification of inactive structures reveals residues associated with kinase functional specificity. To enable classification of new crystal structures, we have made our classifier publicly accessible through a stand-alone program housed at https://github.com/esbg/kinconform [DOI:10.5281/zenodo.249090].

**Electronic supplementary material:**

The online version of this article (doi:10.1186/s12859-017-1506-2) contains supplementary material, which is available to authorized users.

## Background

Protein kinases are a diverse family of signaling proteins whose catalytic activity is involved in nearly all cellular processes. The enzymatic activity of kinases is regulated through conformational changes in the protein kinase domain, which is shared by diverse members of the protein kinase super-family [1–3]. Structural studies on members of the protein kinase superfamily have shown that the protein kinase domain is malleable and can undergo dramatic conformational changes in response to activation and regulatory signals in signaling pathways. The conformation of protein kinases is controlled by factors including protein-protein interactions [4], phosphorylation and ligand binding [5, 6], and numerous drug discovery efforts on protein kinases are focused on targeting specific kinase conformations [7–11]. However, an incomplete understanding of the defining conformational features of protein kinases and how these differ between kinase groups and families has hindered ongoing drug discovery efforts to improve inhibitor specificity.

**Fig. 1:**
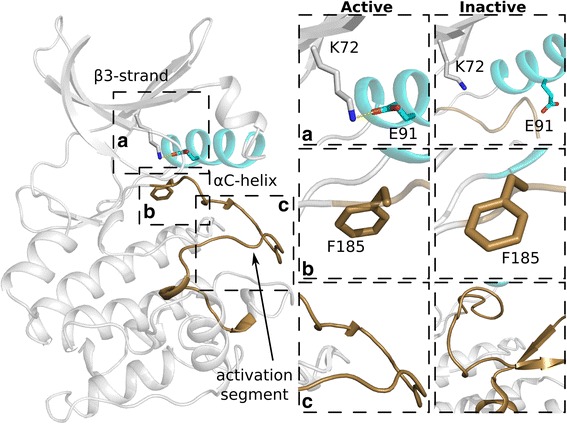
Kinase activation. **a** Formation of the K-E salt bridge (left). The salt bridge is not formed in the inactive conformation (right). **b** DFG-in (left) and DFG-out (right) conformations. **c** The inactive activation segment (right) can occlude the substrate binding region. The αC-helix is colored teal and the activation segment is colored brown

The manual inspection of kinase crystal structures has led to an understanding of the roles key residues play in stabilizing and orienting adenosine tri-phosphate (ATP) for phosphoryl transfer [12, 13], as well as qualitative descriptions of the active site geometry and conformational states. A variety of structural measures have been developed to determine the activation state of a kinase, most of which center on the orientation of two regulatory components: the αC-helix and the activation segment. The αC-helix serves as a proxy for the formation of a catalytically crucial salt bridge between a lysine in the β3 strand and the αC-helix glutamate (K-E salt bridge), which positions the lysine to anchor the α-phosphate of ATP (Fig. 1a). The activation segment provides two pieces of information: the orientation of the DFG aspartate, which chelates a magnesium ion that coordinates with the β- and γ-phosphates of ATP (Fig. 1b), and whether the C-terminal activation segment is blocking the substrate binding site (Fig. 1c).

The orientation of the DFG phenylalanine, a proxy for the DFG aspartate located in the N-terminal activation segment, is of particular importance for inhibitor design, as the DFG-out conformation creates a hydrophobic pocket that can be targeted by Type II ATP competitive inhibitors [7, 8, 14–16]. Studies focused on the classification of active site conformation for drug discovery purposes have generally used manual curation [17] or constructed complex template based metrics specific for that purpose [18]. For example, in Kufareva and Abagyan, the authors chose a template DFG-in conformation in the Abelson tyrosine kinase (Abl1) structure [PDB: 2GQG] to generate a DFG-Phe orientation index, O_phe_, by summing the cosines of angles between covalent bonds (Cα-Cβ, Cβ-Cγ, Cγ-Cδ^1^, Cγ-Cδ^2^) in the template and aligned structures. The position, P_phe_, was calculated as the distance between the Cα atoms in the template and aligned DFG-Phe residue, with the final DFG-in score (S_DFG-in_) defined as a function of O_phe_ and P_phe_. A hard cutoff of S_DFG-in_ was used to classify DFG-in versus DFG-out conformations. Methods that consider activation segment conformation to classify kinase structures have been reported as well. One such method used a comparison to the cAMP-dependent protein kinase catalytic subunit α (PKAα) structure [PDB:1BKX], where root mean squared deviation (RMSD) calculations and counting atoms separated by predefined planes determined the conformational state of the αC-helix and activation segments, respectively, with hard cutoffs distinguishing active from inactive kinases [19]. Other methods have benefitted from exponential increases in published kinase structures, though limited themselves to measuring the αC-helix and DFG motif conformations. In one (Brooijmans’), the distance between the side chain nitrogen of the β3 lysine and the αC-helix glutamate’s terminal oxygen’s were used to measure the orientation of the αC-helix and the K-E salt bridge distance, using a hard cutoff of 4Å. The activation segment orientation was measured using the distance between the main chain nitrogen in the hinge donor residue and the Cα atom in the DFG-Phe, with a soft cutoff of 15Å [20]. In another, the αC-helix orientation (in/out/out-like) was determined using the distance between the Cα atoms of the DFG aspartate and the αC-helix glutamate, while the DFG motif orientation was manually curated [21]. The most recent approach (ABC method) notes that the DFG orientation is captured more accurately with pseudo-dihedral angles, or dihedrals through adjacent quads of Cα atoms [22]. Multiple conformational states for both the DFG motif and the αC-helix are defined, but the active conformation is only attained when both the DFG-in and αC-helix-in orientations are present. The αC-helix orientation is defined using the distance between the Cα atom of the αC-helix glutamate and the Cα atom of the DFG-Phe, with the αC-helix-in conformation occurring when this distance is less than 10.5Å. The DFG orientation is measured using three pseudo-dihedral angles, with the DFG-in state defined when all three pseuo-dihedrals are within specified ranges.

Perhaps the most interesting classification method, as it was the first method published and does not explicitly measure any angles or distances, is the formation of the hydrophobic Regulatory spine (R-spine) [23, 24], discovered through the surface comparison of 23 kinase structures. The formation of hydrophobic interactions between the N- and C-lobes of the kinase domain are described as characteristic of active structures, with inactive structures breaking the spine in four ways [24]. While this method is highly qualitative and difficult to measure, the spine formation is mechanistically explained, with the assembly of the spine coordinated with other conformational changes associated with kinase activation such as the formation of the K-E salt bridge and the relative orientation of the catalytically important DFG and HRD motifs [23]. Though the precise order in which these interactions develop is unknown, the assembly of the R-spine is highly dependent on the orientation of the activation segment [25].

**Fig. 2:**
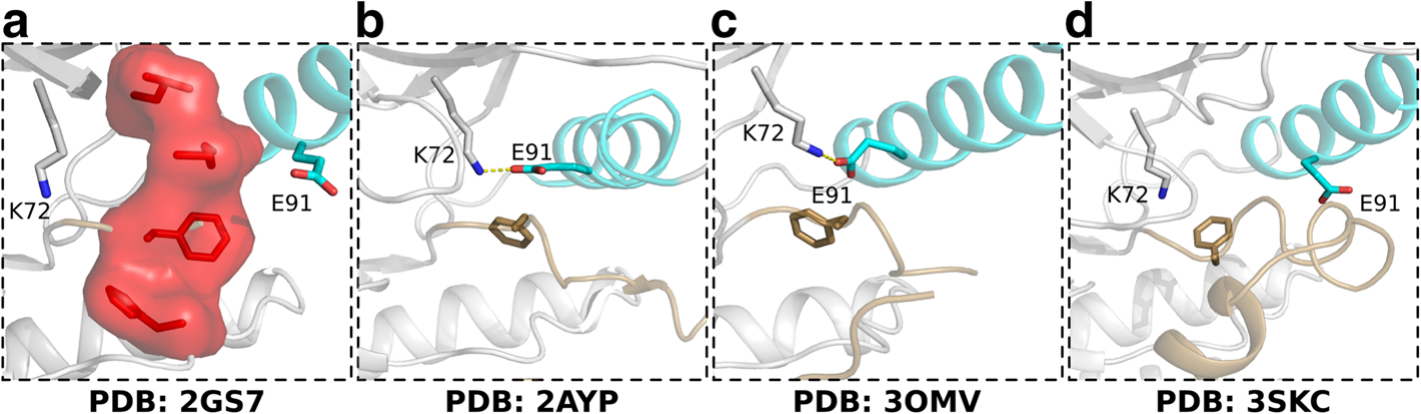
Incorrectly classified structures. **a** Assembled R-spine without K-E salt bridge formed. **b** Structure improperly annotated using Brooijmans’ method. **c**–**d** Structures improperly annotated using ABC method. The αC-helix is colored teal and the activation segment is colored brown

While the above methods have provided insights into kinase conformational states, they are limited in several ways. Many only consider a small subset of the 5,131 kinase chains deposited in the PDB, thereby leaving out valuable information. Some studies have used a large subset of structures/conformations, but only a limited number of features, with emphasis placed on the active site orientation [19–23]. These limitations lead to conflicting assessments and, in some cases, misannotation of kinase conformations. For example, while a broken R-spine is characteristic of an inactive kinase, an assembled R-spine does not necessarily reflect an active conformation. In the epidermal growth factor receptor (EGFR) structure [PDB:2GS7] for example, the R-spine is assembled, but the activation segment is in an inactive conformation with a disrupted K-E salt bridge (Fig. 2a). Distance measures are problematic when the endpoint atoms are in dynamic loop regions. The active checkpoint kinase 1 (Chk1) structure [PDB:2AYP], which has an unusual linker conformation, is incorrectly annotated as inactive using Brooijmans’ method (Fig. 2b). The most recently published ABC method, which uses hard cutoffs on angle measurements, also leads to improper annotations. For example, the RAF proto-oncogene serine/threonine protein kinase (RAF1) structure [PDB:3OMV] is clearly active, with a well-established K-E salt bridge and DFG-in conformation, yet is annotated by ABC as inactive (Fig. 2c). In contrast, the inactive serine/threonine protein kinase B-raf (BRAF) structure [PDB:3SKC] is annotated by ABC as active (Fig. 2d). Further, complex correlations between measurements can be difficult to identify and interpret without an appropriate statistical framework. Here, we take a systematic statistically based approach, using the automated pattern recognition algorithms in machine learning to identify the conformational changes between active and inactive protein kinases. We find that the orientation of the activation segment alone is sufficient to accurately classify kinase conformations as active or inactive, and identify the relative importance of different regions of the activation segment in classifying protein tyrosine kinase (PTK) and serine/threonine kinase (STK) conformations. We show the greatest variation between inactive structures results from evolutionary relationships between kinases, identifying a variety of residues that can be used to increase drug specificity. Finally, by applying our methods to the cyclin-dependent kinase family (CDKs), we identify interface residues associated with cyclin binding and recognition. While machine learning methods have been widely used in secondary structure and backbone torsion angle prediction from primary sequence [26–29], their use in structure based classification tasks has been limited to the identification of Structural Classification of Proteins (SCOP) domains and ligand prediction [30, 31]. Only recently have machine learning techniques been applied to identify quantitative structure-activity relationships (QSARs) [32] or improve protein homology detection [33], though even these techniques are limited to using primary sequence input. When structures are used in machine learning algorithms, they are typically subjected to molecular dynamics simulations which are used to calculate a parameter of interest, such as energy landscapes [34] or thermostability [35]. We also provide access to the established classifier through a stand-alone program, allowing any user to classify PDB structures or models, and have deposited our training annotations and predictions in ProKinO, the Protein Kinase Ontology [36, 37].

## Results & discussion

### Kinase conformation is determined by activation segment orientation

**Fig. 3:**
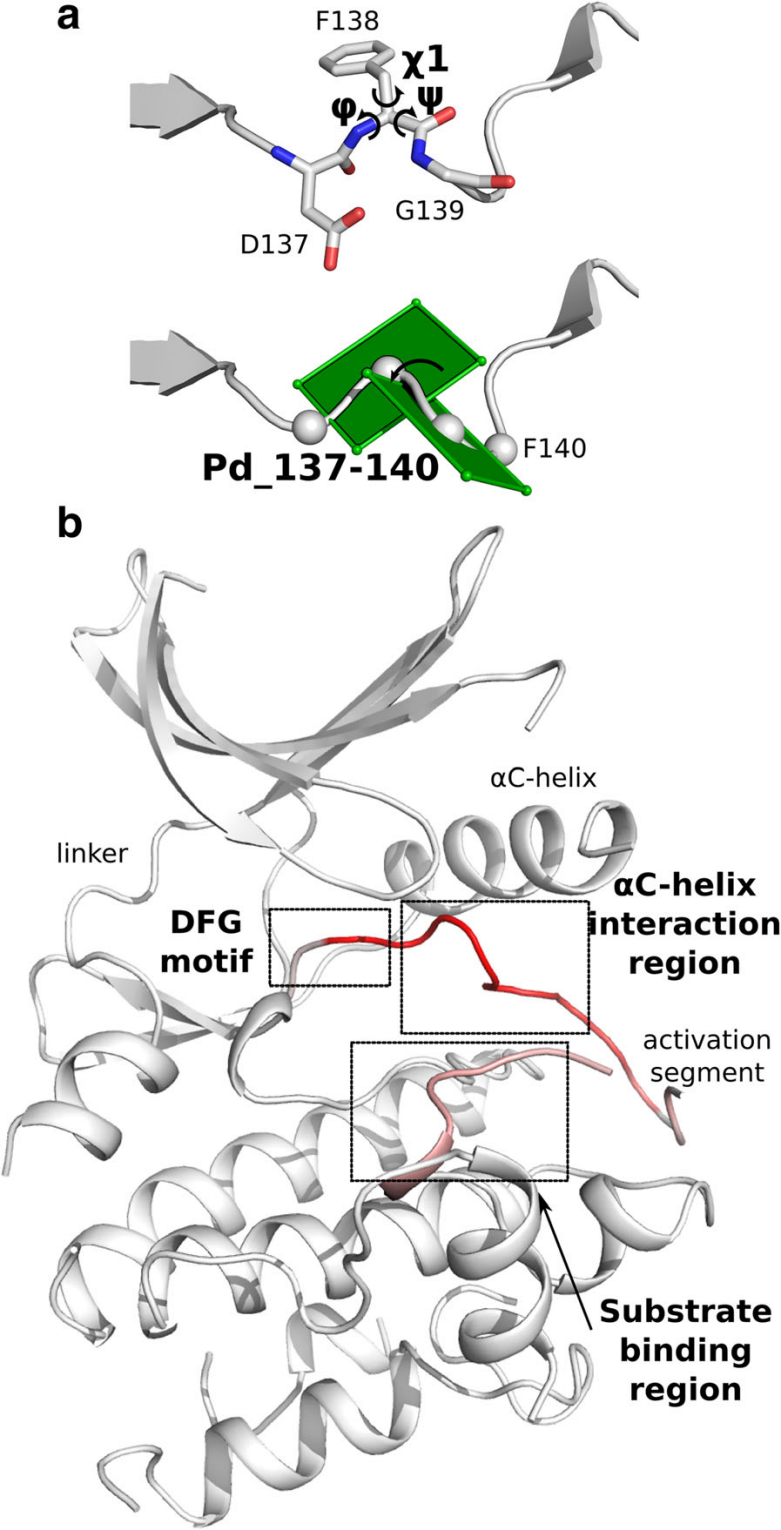
ePK features. **a** Features measured for classification. For each aligned residue, we calculate the φ, ψ and χ1 angles, depicted for residue 138 (top). In addition, for quads of adjacent residues, we calculate the pseudo-dihedral through their Cα atoms, with Pd_137-140 shown (bot). **b** Selected features on template structure colored by importance from white (no weight) to red (high weight)

Previous methods have focused on the active site geometry for conformation classification, using the DFG motif and αC-helix orientations as proxies for the catalytically necessary placement of key residues. This active site focus naturally biases the proposed measurements by ignoring distal regions of the kinase domain. Instead, we developed features that encompass the entire domain and uniquely define each structure. Namely, for each residue in our profile based alignments, we incorporate the corresponding φ, ψ and χ1 angles. In addition, for each set of four consecutive residues, we calculate the pseudo-dihedral between their Cα atoms (Fig. 3a). Using our feature selection process on the described training set (see Methods), we rank ordered the 961 features, the top 15 of which are identified in Table 1. The regions identified are highly consistent between selection algorithms and identify measures describing the orientation of the activation segment. We divided the activation segment into three sections, based on their function and interacting regions. The most N-terminal portion describes the orientation of the DFG motif, followed by a region which forms electrostatic and hydrophobic interactions with the αC-helix, and finally the most C-terminal portion of the activation segment, which can sterically block the binding of substrate (Fig. 3b).

**Table 1:** Top 15 ePK selected features

Feature	Activation segment location	PKA positions	Average rank
Pd_137-140	N-terminal	184–187	2.88
Φ_141	N-terminal	188	4.20
Pd_140-143	N-terminal	187–190	7.95
Pd_141-144	N-terminal	188–191	8.95
Φ_142	N-terminal	189	9.57
Φ_143	N-terminal	190	10.15
Ψ_139	N-terminal	186	10.65
Φ_138	N-terminal	185	14.15
Pd_139-142	N-terminal	186–189	20.75
Pd_155-158	C-terminal	200–203	22.15
Pd_138-141	N-terminal	185–188	27.00
Pd_135-138	N-terminal	182–185	27.25
Pd_136-139	N-terminal	183–186	30.02
Pd_154-157	C-terminal	199–202	31.02
Φ_142	N-terminal	189	32.20

**Fig. 4:**
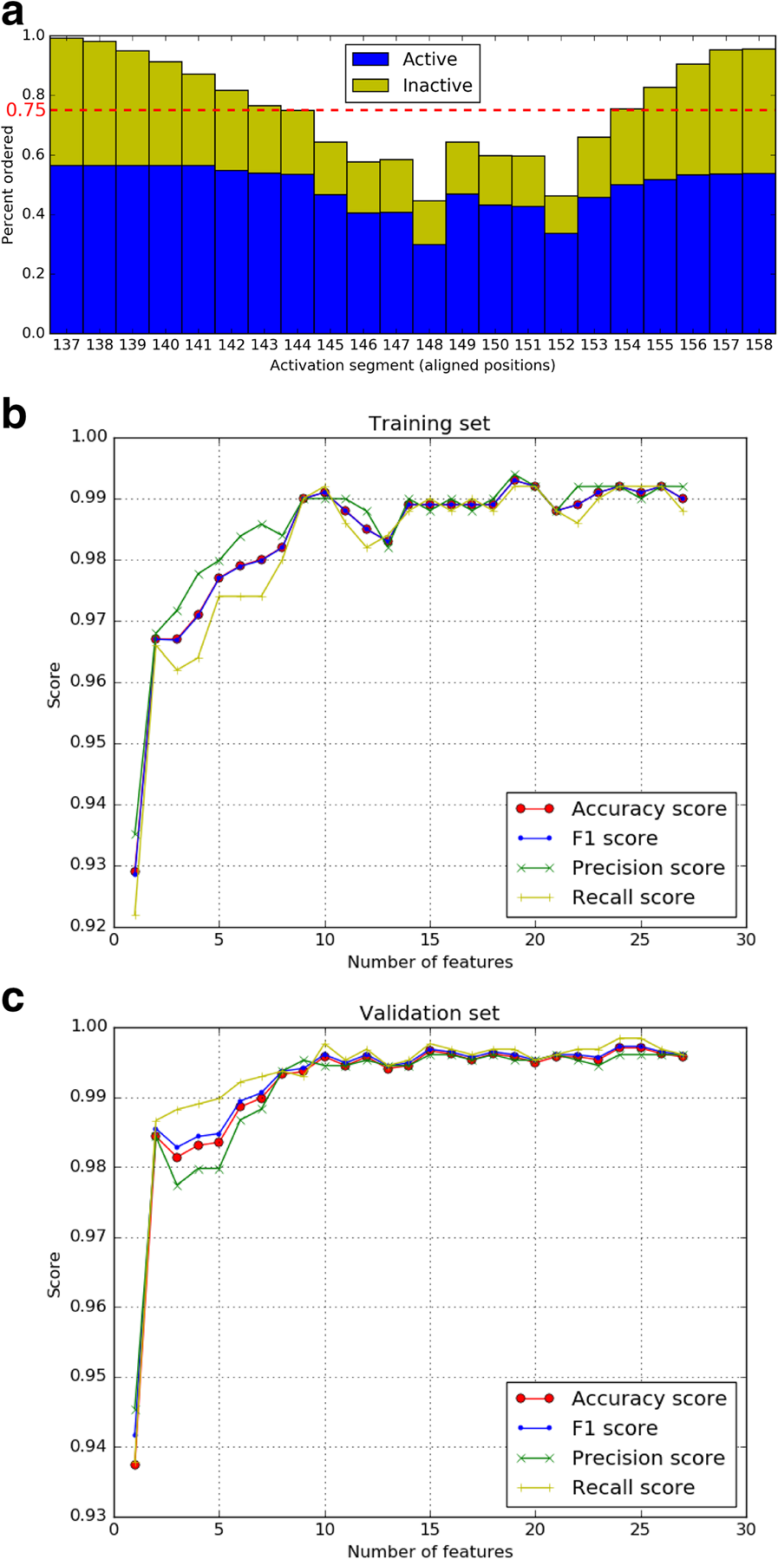
Disorder and accuracy. **a** Chart showing the percentage of structures with activation segment disorder. **b** Accuracy with increasing number of features on training set. **c** Accuracy with increasing number of features on validation set

As coordinates of residues in the activation segment are commonly missing from crystal structures, regardless of conformation, we identified the frequency of activation segment residues without missing coordinates across the full dataset (Fig. 4a). To ensure we were not simply capturing the presence/absence of coordinates as a feature for classification, we only considered residues (and associated features) if they were present in more than 75% of the dataset. To assess the relative importance of the selected features, we initially trained a random forest classifier based on the most frequently chosen feature, which captures the orientation of the DFG motif. Using this feature alone, we achieved over 90% classification accuracy, with precision and recall slightly higher and lower, respectively (Fig. 4b–c). The addition of the second feature, which describes the orientation of the activation segment following the DFG motif, increased our classification accuracy to ~97%. Subsequently, we trained additional classifiers with an increasing number of features, evaluating the accuracy increase associated with the incorporation of each new feature on classifying our training (Fig. 4b) and validation (Fig. 4c) sets.

**Fig. 5:**
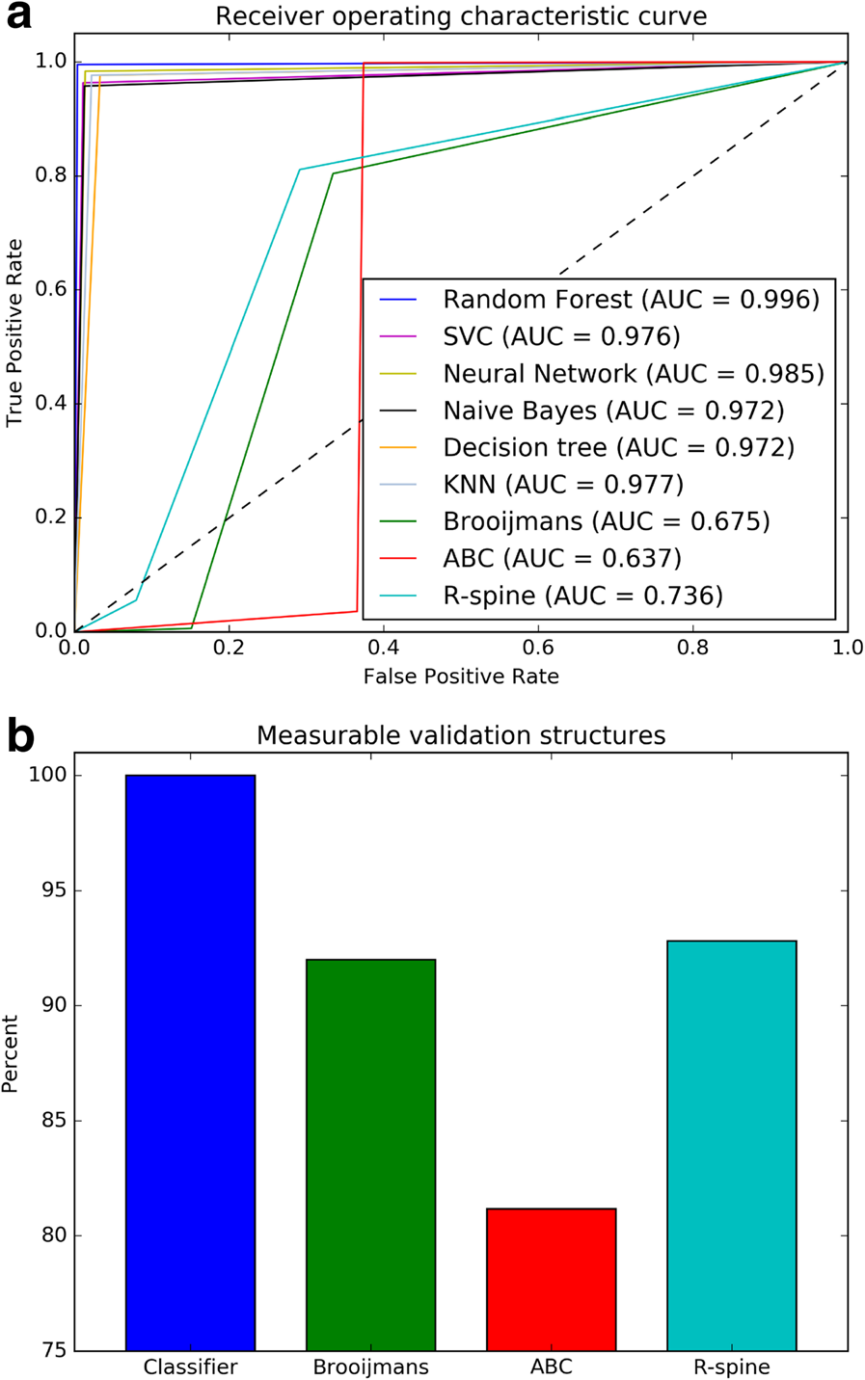
**a** Receiver operating characteristic curve comparing classifiers and previous methods. **b** Percent of structures which can be measured and classified with various approaches

We compared the performance of a variety of machine learning classifiers, the Brooijmans’, ABC, and R-spine methods using our validation set. As the criteria for determining the assembly of the R-spine is not well defined, we calculated the van der Waals (vdW) interactions between pairs of residues which are adjacent in the spine. If the vdW energy is less than -1.5 kcal/mol, we consider the spine to be assembled. This approach accurately identifies previously noted R-spine assembled structures [23]. Of the previously defined methods, the R-spine assembly has the highest area under the curve (AUC) (0.736) and is able to classify the largest proportion of structures, missing only 6.7% (158 of 2,365 chains) of the validation set. The ABC method performs well when the necessary residues are ordered, but is unable to classify 18.8% (445 of 2,365 chains) of the validation set. The Brooijmans’ method has lower performance when it is able to make an assessment, but only misses 8.2% (193 of 2,365 chains) of the validation set. Our machine learning classifiers, however, can assess 100% of the validation set, with all algorithms achieving classification accuracies greater than 97% (Fig. 5a). Furthermore, we correctly classify the EGFR [PDB:2GS7], Chk1 [PDB:2AYP], RAF1 [PDB:3OMV], and BRAF [PDB:3SKC] structures which were among those incorrectly annotated using the previous methods. While the generated classifiers can accurately predict the conformation of all the structures in our validation set, previous methods cannot make a prediction with missing atoms or residues (Fig. 5b). We chose the ensemble random forest classifier for the remainder of analyses, as it out performed all other algorithms.

**Fig. 6:**
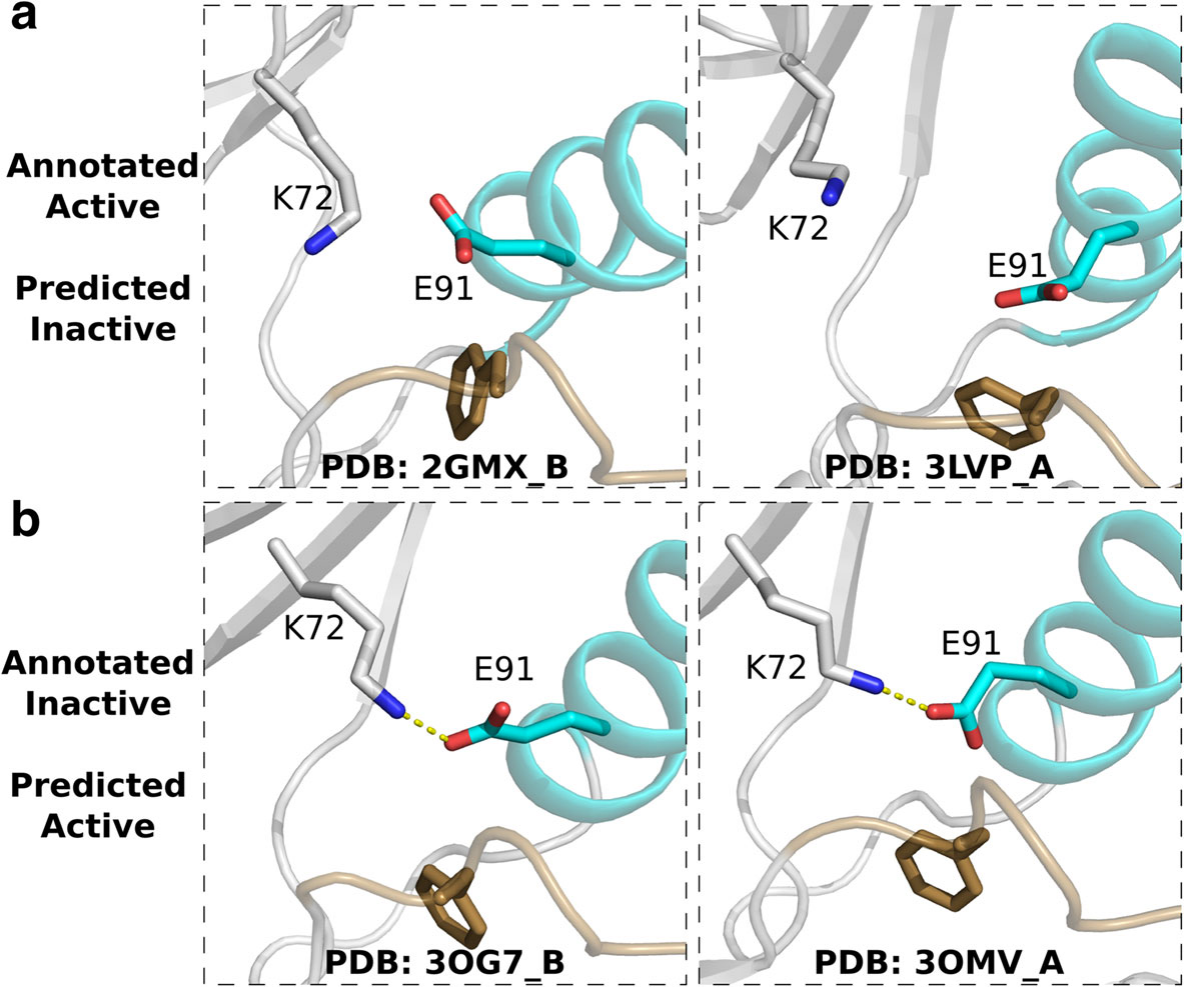
Misannotated structures. **a** Structures misannotated as active which were properly identified as inactive by the classifier. **b** Structures misannotated as inactive were properly classified as active by the classifier. The αC-helix is colored teal and the activation segment is colored brown

Modification of the activation segment by phosphorylation is an important step in kinase activation and is required for most kinases to become fully active [6]. The conformational changes brought about by the phosphorylation event, which include ordering of the C-terminal activation segment for substrate binding, have been previously described [38–40]. The C-terminal activation segment, however, has not been used to assess kinase conformational state. Our identification of the N-terminal activation segment is consistent with previous classification models, particularly with our top selected feature which relates the orientation of the DFG-Phe to the conformational state, but also incorporates previously unidentified information concerning the orientation of the C-terminal activation segment. Nearly all validation structures were properly classified, leaving only 12 PDB chains whose curated annotation differed from the predicted conformational state. Manual examination of the 12 chains indicated that the difference is not due to machine learning classifier, but rather due to the misannotation of chains in the training set (Fig. 6). The chains are part of homo-dimeric complexes where one chain adopts an active conformation, and the other an inactive conformation, however, both chains were annotated as active or inactive based on manual evaluation of a single chain. For example, in the Mitogen-activated protein kinase 8 (JNK1) [PDB: 2GMX] in chain A, the K-E salt bridge is assembled (active), but not in chain B (inactive). However, both chains were annotated as active based on examination of chain A alone. Thus, the 12 chains were not actually misclassified, but rather the classifier had identified errors in our curated annotations.

### K-E salt bridge formation is dependent on activation segment orientation

Our classifier was trained using features specific to the orientation of the activation segment, a deviation from previous methods which also incorporated αC-helix orientation and the formation of the K-E salt bridge. The orientation of the loops preceding and following the αC-helix were captured in our initial feature set, providing information about the αC-helix orientation, but weren’t identified by feature selection as important for classification. This suggests the αC-helix orientation, and hence the salt bridge formation, is dependent on the orientation of activation segment, which is captured in our selected features. To test this hypothesis, we used our trained ePK classifier to predict the conformational state of the remaining 1,766 unlabelled structures. As the features used correspond to the activation segment, we are essentially using only that portion of the kinase domain to distinguish active from inactive kinases. We also measured the distance between the side chain nitrogen atom in the beta-3 lysine and oxygen atoms in the αC-helix glutamate of the same structures. If the distance was less than 4Å, we classified the structure as having an intact salt bridge (Table 2). We identified statistical correlations between the active-inactive classification and salt bridge formation, using both Fisher’s exact and chi-squared tests. We find a strong dependence relationship between activation segment orientation and formation of the K-E salt bridge, with a *p*-value on the order of 10^-49^, explaining the lack of identified features measuring αC-helix orientation. We do not need to measure the αC-helix separately, as we gain the information by considering only the activation segment orientation.

**Table 2:** Testing dependence of activation segment orientation and formation of the K-E salt bridge

	K-E bridge	No K-E bridge
Predicted active	846	205
Predicted inactive	200	278

*P*-value < 10^-49^ with Fisher’s exact and chi-squared tests

**Fig. 7:**
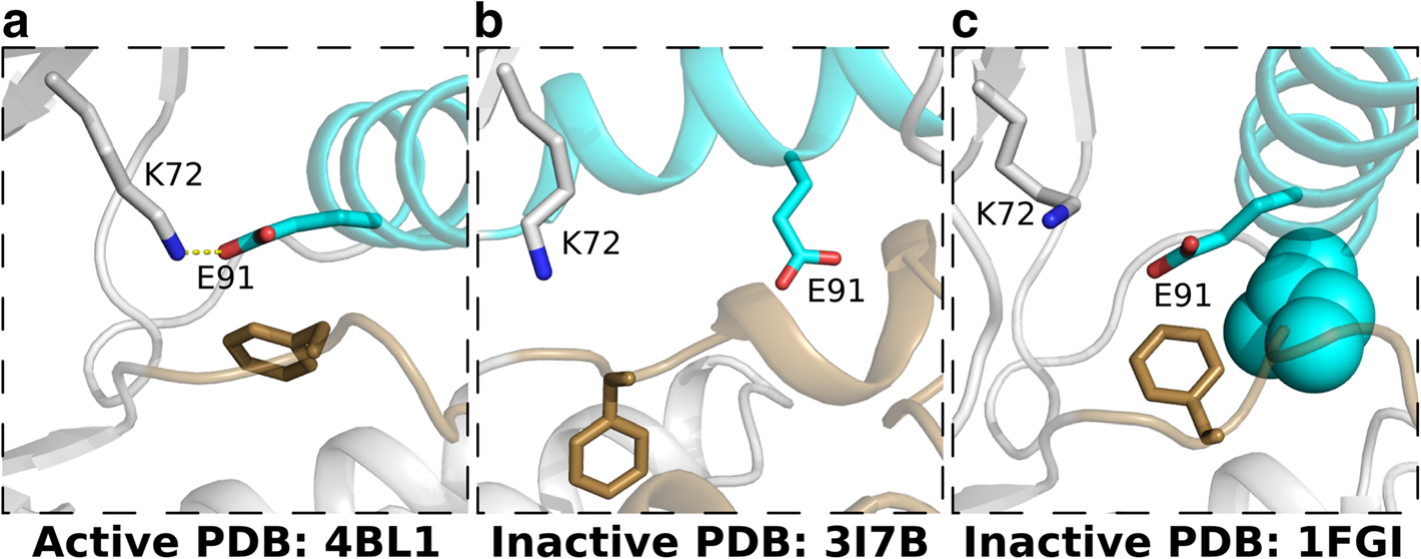
Activation segment conformations. **a** Active conformation. **b** Inactive conformation with 1 ½ turn activation segment helix sterically blocking αC-helix movement. **c** Inactive conformation with DFG-Phe up sterically blocking formation of K-E salt bridge. The αC-helix is colored teal and the activation segment is colored brown

We can also identify the αC-helix/activation segment dependence through the visual inspection of crystal structures. When the kinase is in an active conformation, the DFG-phenylalanine adopts the in-conformation and the activation segment is pulled back, allowing the αC-helix to move in towards the active site and form the K-E salt bridge with the β3 lysine (Fig. 7a). However, several inactive conformations of the activation segment can hinder αC-helix movement. For example, the activation segment of inactive structures often forms a 1 ½ turn helix C-terminal to the DFG motif, while the αC-helix is moved away from the active site, rotating the conserved glutamate towards the solvent (Fig. 7b). Here, the 1 ½ turn helix makes electrostatic and hydrophobic interactions with the displaced αC-helix, stabilizing the inactive conformation. Alternatively, the DFG-phenylalanine can adopt an up-conformation, which only slightly hinders αC-helix movement yet sterically blocks the formation of the K-E salt bridge. In this situation, the αC-helix visually appears to be in the in-conformation and aligning to active structures shows little difference (Fig. 7c). It should be noted that K-E salt bridge formation does not guarantee an active kinase, as the salt bridge can be formed in the DFG-out inactive conformation.

### PTK and STK conformations are distinguished by different regions of the activation segment

**Fig. 8:**
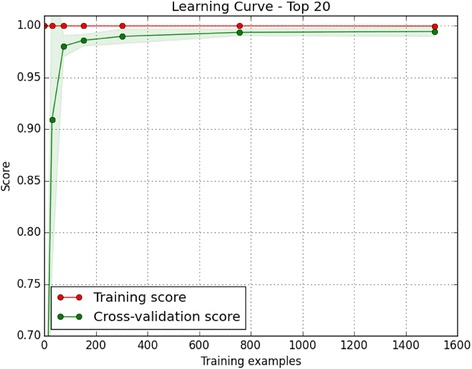
Learning curve on ePK classifier. Learning curve on the ePK classifier for the top 20 selected features. With more than 200 training samples, we achieve high classification accuracy

Next, we wanted to apply our approach to evolutionarily related subsets of kinases. However, the number of solved structures is not uniformly distributed across the kinome, and some groups, families and subfamilies are not well represented. To ensure meaningful results, we first identified the number of training samples needed to construct an accurate classifier through the generation of learning curves, which train classifiers using an increasing number of data points and assesses the accuracy of each classifier. Fewer than 200 structures were needed as training data to classify the remainder with greater than 99% accuracy (Fig. 8). As we have 1,008 labeled protein tyrosine kinase (PTK) and 2,357 serine-threonine kinase (STK) chains, we applied our feature selection process to these sets.

**Fig. 9:**
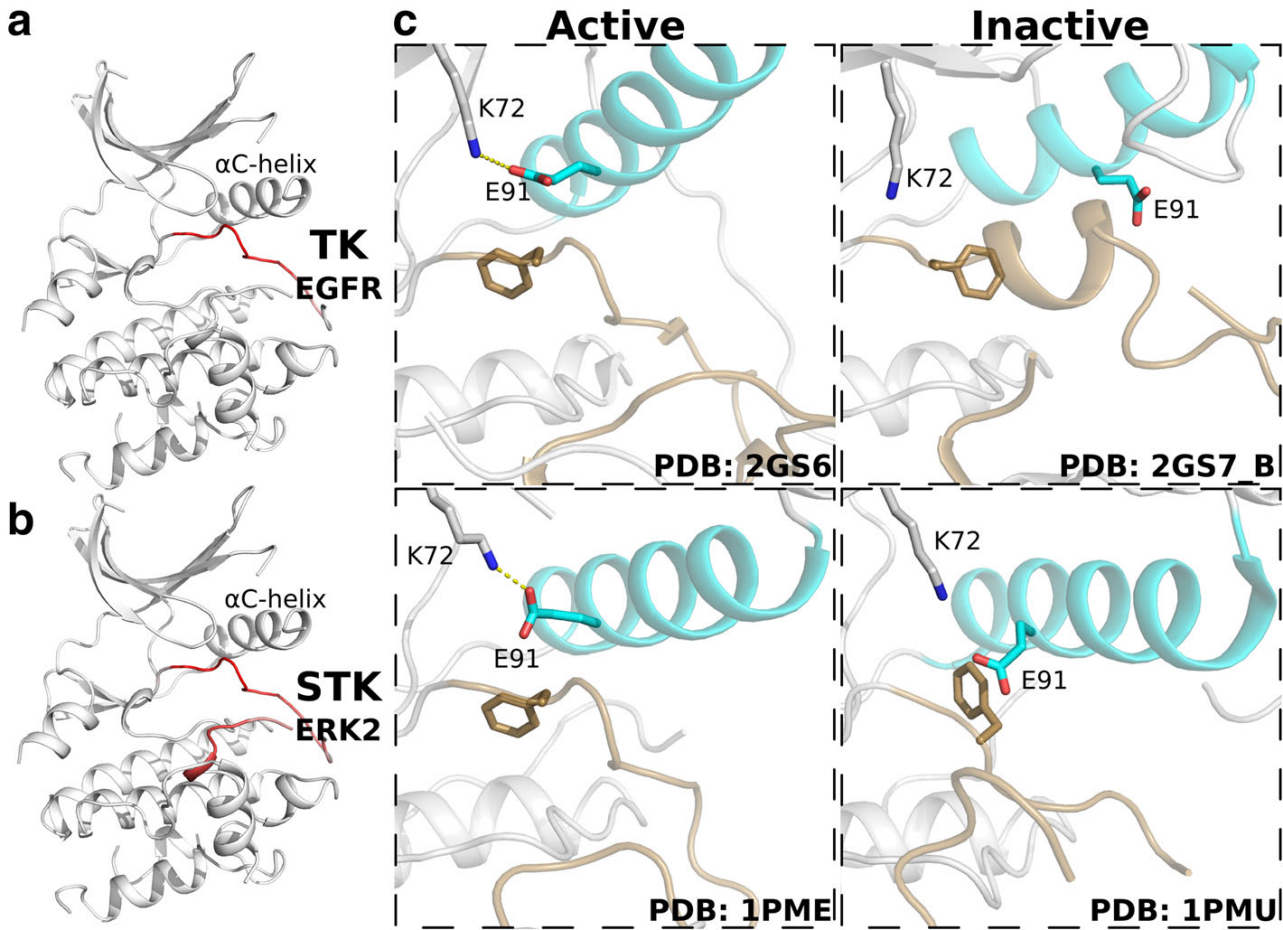
PTK/STK features (**a**) PTK selected features. **b** STK selected features. **c** Structures of the active (left) and inactive (right) conformations observed in PTKs (top) and STKs (bottom). The αC-helix is colored teal and the activation segment is colored brown

In PTKs, we achieve a stable >99% classification accuracy with the top 10 selected features (Table 3), all of which occur in the N-terminal activation segment (Fig. 9a). STK classifiers, on the other hand, do not achieve stability in classification accuracy until ~20 features are incorporated (Table 4), with important C-terminal activation segment features identified (Fig. 9b). To ensure our STK measures are not dominated by a single group, since 46% (1,090 of 2,357) of our STK chains belong to the CMGC group, a collection of kinases named after its four major members – the CDK, MAPK, GSK3, and CLK families, we also performed feature selection on the non-CMGC STKs. Again, the top selected features corresponded to both the N- and C-terminal activation segments (Table 5). This is consistent with the role of activation dependent phosphorylation in the N-terminal activation segment of both PTKs and STKs, but suggests that the orientation of the C-terminal activation segment is more informative in the conformational classification of STKs.

**Table 3:** Top 10 PTK selected features

Feature	Activation segment location	PKA positions	Average rank
Pd_140-143	N-terminal	187–190	3.10
Pd_137-140	N-terminal	184–187	3.40
Φ_141	N-terminal	188	8.75
Pd_141-144	N-terminal	188–191	11.08
Ψ_139	N-terminal	186	11.60
Φ_143	N-terminal	190	13.42
Φ_142	N-terminal	189	14.90
Pd_139-142	N-terminal	186–189	14.98
Pd_138-141	N-terminal	185–188	25.70
Pd_135-138	N-terminal	182–185	28.70

**Table 4:** Top 20 STK selected features

Feature	Activation segment location	PKA positions	Average rank
Pd_137-140	N-terminal	184–187	3.20
Ψ_141	N-terminal	188	6.97
Ψ_142	N-terminal	189	8.85
Pd_140-143	N-terminal	187–190	11.25
Pd_155-158	C-terminal	200–203	13.78
Pd_141-144	N-terminal	188–191	15.07
Ψ_138	N-terminal	185	15.23
Φ_139	N-terminal	186	17.13
Ψ_144	N-terminal	191	20.45
Ψ_143	N-terminal	190	21.98
Pd_139-142	N-terminal	186–189	29.35
Φ_155	C-terminal	200	30.13
Pd_138-141	N-terminal	185–188	32.80
Pd_136-139	N-terminal	183–186	33.05
Φ_142	N-terminal	189	37.57
Pd_135-138	N-terminal	182–185	38.42
Ψ_139	N-terminal	186	43.98
Ψ_154	C-terminal	199	46.17
Pd_156-159	C-terminal	201–204	48.70
Ψ_157	C-terminal	202	49.08

**Table 5:** Top 20 non-CMGC STK selected features

Feature	Activation segment location	PKA positions	Average rank
Pd_137-140	N-terminal	184–187	5.30
Pd_155-158	C-terminal	200–203	15.23
Pd_136-139	N-terminal	183–186	19.77
Φ_155	C-terminal	200	27.30
Pd_141-144	N-terminal	188–191	27.98
Pd_139-142	N-terminal	186–189	28.90
Φ_139	N-terminal	186	34.55
Ψ_139	N-terminal	186	34.83
Ψ_141	N-terminal	188	38.37
Pd_140-143	N-terminal	187–190	40.02
Ψ_144	N-terminal	191	40.65
Pd_135-138	N-terminal	182–185	42.88
Pd_138-141	N-terminal	185–188	44.73
Ψ_154	C-terminal	199	47.73
Φ_137	N-terminal	184	48.65
Φ_155	C-terminal	200	49.75
Φ_154	C-terminal	199	53.75
Ψ_143	N-terminal	190	59.55
Ψ_138	N-terminal	185	61.13
Ψ_156	C-terminal	201	65.42

We can observe the differences in activation segment conformations in PTK and STK crystal structures (Fig. 9c). In the PTK EGFR, for example, we observe large conformational changes in the N-terminal activation segment, with the 1 ½ turn helix blocking the αC-helix in the inactive conformation. However, only slight changes can be seen in the C-terminal activation segment of EGFR between the active and inactive conformations. In contrast, in STK structures such as ERK2, the N-terminal activation segment is less drastically altered than in EGFR, with only a slight rotation in the DFG-phenylalanine. The ERK2 inactive C-terminal activation segment is markedly changed between the active and inactive conformations, however, with an uncoiled helix that partially blocks active site access. The necessity of incorporating C-terminal activation segment features in STK classification may be due to the fact that STKs conserve phosphorylatable residues in the C-terminus of the activation segment [41–52]. In contrast, PTKs naturally conserve hydrophobic residues at the activation segment C-terminus, providing a plausible explanation for the observed difference in PTK and STK features.

### CDK family feature selection identifies cyclin binding residues

**Fig. 10:**
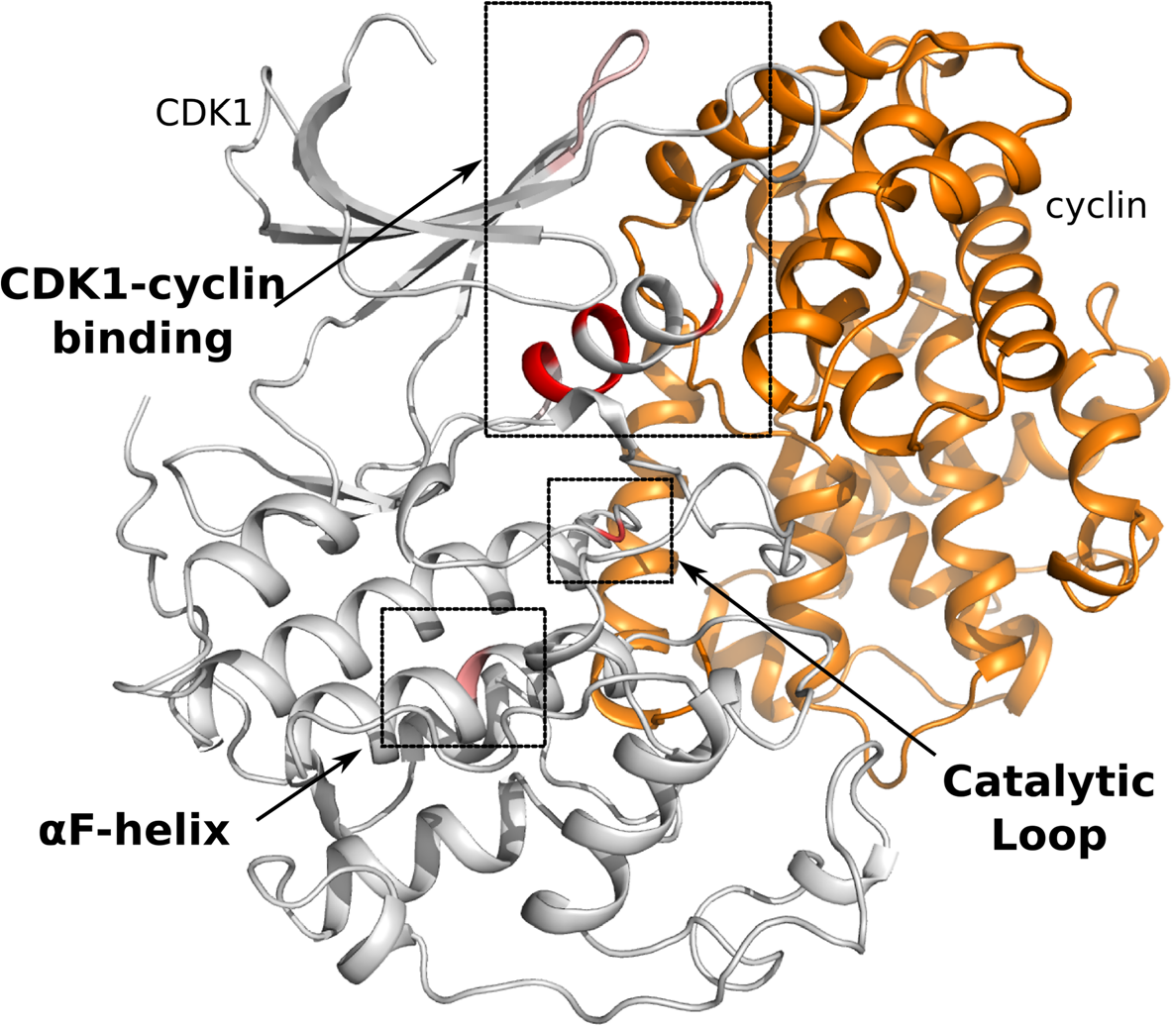
CDK1 with selected features. We identify features in three regions: the cyclin binding interface, the catalytic loop, and the αF-helix. CDK1 is colored white, with identified features in red. Cyclin is colored orange

The cyclin-dependent kinase (CDK) family is a set of STKs involved in cell-cycle progression [53], replication stress response [54, 55] and transcription [56, 57]. CDK activity is, as its name suggests, dependent on the formation of a CDK-cyclin complex. Upon binding, cyclin induces conformational changes in the kinase domain that allow for autophosphorylation of the activation segment to produce a fully active kinase [39]. The CDK family also has the largest number of crystal structures deposited in the PDB, providing 514 chains for our feature selection process. As we previously described, STK (and thus, CDK) classification is based on both N- and C- terminal activation segments. However, CDKs are allosterically regulated through cyclin binding. To test whether our feature selection procedure would be able to identify the CDK-cyclin interactions, we removed the activation segment features from our CDK training samples and repeated feature selection on this subset. The most important features are found in the β3-αC loop and the C-terminal segment of the αC helix, both of which are involved in the CDK-cyclin interface. Less significant features in the CDK-cyclin interface were identified in the β4-β5 loop. Two additional features were found that are distal to the cyclin binding site, one in the catalytic loop and the other in the αF helix (Fig. 10).

### Inactive structures cluster by evolutionary history

To identify patterns in the conformation of inactive structures we took an unsupervised approach, using principal component analysis (PCA) to identify the direction of greatest variance in our dataset. As PCA is an inherently linear process, we transformed the cyclical angle measures of the inactive structures to the Cartesian coordinates on the unit circle (θ → (sin θ, cos θ). After limiting the dataset to the top weighted features in the first three principal components, we again performed PCA to ensure the clustering patterns were similar.

**Fig. 11:**
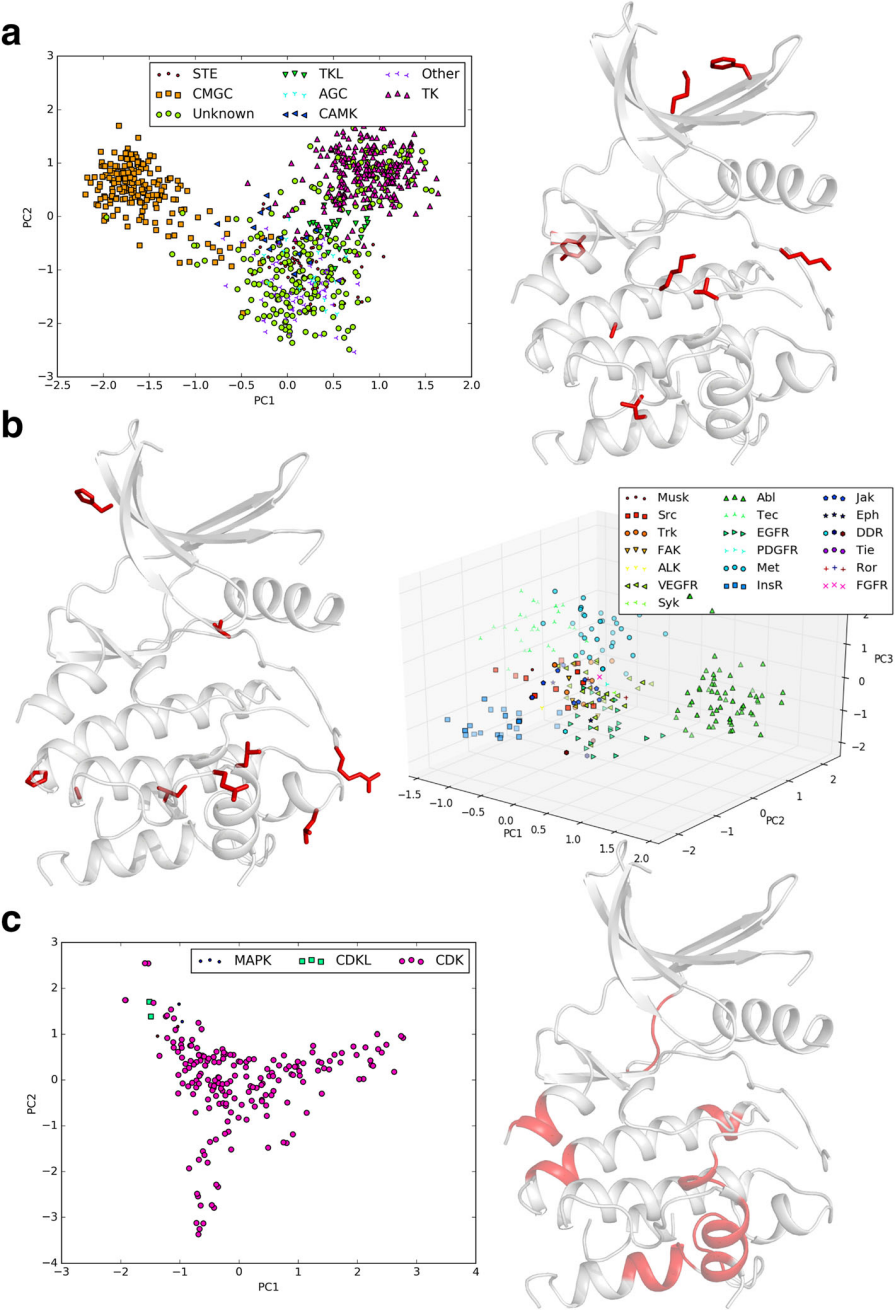
Inactive clusters and features. **a** All ePKs. **b** PTK group. **c** CMGC group

When we applied the above process to all ePK inactive structures, we discovered that the most heavily weighted features were not measuring backbone conformations with φ, ψ, or pseudo-dihedral angles, but χ1 angles which measure side chain orientation (Table 6). The residues identified are spread throughout the kinase domain, occurring in both the N- and C- lobes, with several residues identified in the catalytic loop and activation segment. By plotting the structures in the principal component plane (PC1, PC2), we readily identified three clusters. Two of the clusters are kinase group specific, separating PTKs and CMGCs from the remainder of kinases in the third cluster (Fig. 11a). This is consistent with group biases of several of the top weighted residues, like the Lys in the LKPEN STK specific motif, which is naturally conserved in PTKs as LAARN. Similarly, the STK specific phosphorylation site at 201^PKA^, which is conserved in PTKs as a hydrophobic residue, is heavily weighted in the principal components.

**Table 6:** Top 10 features in inactive structures among all ePKs, PTKs, and CMGCs

ePK features	PKA positions	TK features	PKA positions	CMGC features	PKA positions
χ1_121	168	χ1_166	211	Pd_217-220	268–271
χ1_64	110	χ1_94	141	Pd_56-59	102–105
χ1_79	125	χ1_197	242	Pd_157-160	202–205
χ1_142	189	χ1_26	68	Pd_202-205	247–250
χ1_100	147	χ1_150	195	Pd_219-222	270–273
χ1_218	269	χ1_235	291	Pd_229-232	280–283
χ1_29	71	χ1_187	232	Pd_109-112	156–159
χ1_132	179	χ1_160	205	Pd_95-98	142–145
χ1_156	201	χ1_147	N/A	Pd_199-202	244–247
χ1_131	178	χ1_52	98	Pd_85-88	131–134

A similar pattern emerged when we considered only the PTK inactive structures. Again, we identify side chain conformations (χ1 angles) as providing the greatest variance in the dataset. Here, the residues identified lie mostly in the C-lobe, occupying positions in the substrate binding region, P + 1 loop, and the activation segment (Table 6). Plotting the structures in the first three principal components reveals an analogous pattern, with clusters consisting of specific PTK families. The most distinct clusters separate the Abelson tyrosine-protein kinase (Abl) family, the Insulin receptor (InsR) family, the tyrosine-protein kinase Tec (Tec) family, and the hepatocyte growth factor receptor (Met) family (Fig. 11b). In contrast, the top weighted features in the CMGC group consist entirely of pseudo-dihedral angles (Table 6). The CMGC inactive structures are largely dominated by the CDK family, likely explaining the variance present in backbone measurements. Again, the features are mostly in the C-lobe, occurring in the αD-helix, at the terminal ends of the αE-helix, and the αG- and αH- helices. The only N-lobe feature identified is in the C-terminal portion of the αC-β4 loop (Fig. 11c). To avoid issues with the side chain placement of low resolution structures, we repeated the above inactive clustering with only high resolution structures (<2.2Å). Again, we find that the greatest source of variance in our inactive datasets correlates with the evolutionary relationships between kinases, with clusters similar to those in Fig. 11. Thus the conformational features appear to implicitly capture the evolutionary relationships between kinases, even though evolutionary features (sequence similarity, for example) are not explicitly considered in our analysis.

### Classifier available for public use

To encourage consistency in kinase structure annotations, we have made our ePK random forest classifier publicly available through a stand-alone program located at https://github.com/esbg/kinconform [DOI:10.5281/zenodo.249090], allowing users to annotate newly solved kinase PDB structures and/or simulated structures as active/inactive. The sequence for each chain is extracted and aligned to our highly curated profile alignments. The features necessary for classification are then measured and a prediction is generated. We have also included both our curated annotations for the training and validation sets, as well as predicted annotations on all kinase PDBs in the structure class of ProKinO, the Protein Kinase Ontology [36, 37]. Using the *hasAnnotatedConformation* and *hasPredictedConformation* data properties, users can easily identify which structures were used for training/validation, which structures were misclassified, as well as the predicted conformation for remaining structures. The dataset of measurements, predicted conformations and annotated conformations are included [see Additional files 1 and 2].

## Conclusion

We have provided the first comprehensive machine learning based classification of protein kinase active/inactive conformations, taking into account more structures and measurements than any previous classification effort. The features identified in our analysis reflect previous knowledge about the conformation of the N-terminal activation segment, as well as provide new insights into the importance of the C-terminal activation segment in classifying STK structures. Given a sufficient number of evolutionarily related structures, we can also identify protein interfaces and regulatory regions, as shown with the cyclin-CDK1 complex. We show statistically significant correlations between activation segment orientation and αC helix orientation, and suggest a classification scheme based on activation segment orientation alone. Further, our unbiased exploration of inactive structures has revealed that the greatest variation between inactive conformations lies in kinase group and family specific side chain orientations. This is interesting given that the evolutionary relationships between kinases are not used as features in the classification.

While we strive to ensure the accuracy of our alignment profiles, the above analysis may be affected by alterations therein. Our methods are also highly dependent on the number of structures available, which is constantly increasing, and the initial set of annotations in the training set. In the future, semi-supervised methods may be beneficial in extending our initial set of curated annotations.

The techniques used above are not kinase specific, and can be applied to any protein family with a conserved fold, a sufficient number of deposited crystal structures, and a curated multiple sequence alignment. Further, while we explored the difference between active and inactive structures, the annotations provided could range over a variety of topics including, whether a ligand, substrate or regulatory molecule is bound (or unbound), the presence (or absence) of a post-translational modification, or any binary attribute of interest. Finally, the residues identified in our analysis can be used in the design of selective protein kinase inhibitors.

## Methods

### Dataset construction

We identified kinase structures in RCSB [58] through sequence alignment to a set of previously generated manually curated kinase profiles [59], yielding 3,488 PDBs with 5,131 chains. To establish our active/inactive annotations, we first classified each of the structures using previously published classification methods. They agreed on the conformational state for 3,098 of the 5,131 chains (60.4%), which we labeled accordingly. Disagreements were settled through consensus manual curation by two independent biochemists, which resulted in sets of labeled and unlabeled chains, with 3,365 and 1,766 members, respectively. We further separated our labeled chains into two sets: a randomly selected training set of 1,000 chains (500 active, 500 inactive) used for feature selection, and a validation set containing the remaining 2,365 chains. This process is quite robust and was repeated 10 times with essentially identical features and classification accuracy. Given the small number of training samples needed to construct an accurate classifier (Fig. 8), one could also perform the initial annotation by selecting and manually curating structures randomly until a balanced dataset of sufficient size is generated.

### Feature construction

For each chain, we created a unique vector which represents the conformation by measuring, the φ, ψ and χ1 angles at each aligned residue in our profile, creating 241*3 = 723 features. In addition, we measured the pseudo-dihedral angle through the alpha carbon of adjacent quads of residues [22], incorporating an additional 238 features and bringing our total feature count to 961. We do not consider pairwise distances between residues, as they would incorporate an additional 28,920 features, and greatly increase the likelihood of over fitting. Measurements were made using the MDAnalysis toolkit [60].

### Feature selection

Feature selection was performed on our training set, consisting of 1,000 PDB chains with an average resolution of 2.2Å. We used a variety of feature selection algorithms (OneR [61], chi-squared, ReliefF [62], Gain-Ratio [63], correlation-based feature selection [64]) with 10-fold cross validation to identify which of the 961 incorporated features are most informative in separating active and inactive structures. The four single-attribute evaluators provide a rank for each feature importance, which we averaged over all evaluators. As our features are numbered according to our profile alignment, we also mapped the identified features onto a template structure to identify their location within the kinase domain. Feature selection was performed with Weka v3.6.11 [65].

### Classifier construction and parameter optimization

We used multiple classification algorithms to classify active from inactive structures, including naïve Bayes, neural network, random forest and support vector classifiers. Naïve Bayes classifiers are probabilistic classifiers which assume independence between the features and apply Bayes’ theorem. Neural networks, inspired by biological neurons, consist of a collection of nodes (neurons) and edges (axons) that are trained on the input data. Support vector classifiers are non-probabilistic and identify the hyper-plane that best partitions the high dimensional space in which the dataset resides. The most accurate classifier was generated using a random forest, which is an ensemble method utilizing a parameterized number of decision trees and outputting their mode as the classification. Each tree in the forest also uses a parameterized maximum number of features in making its decision. We performed a grid search of the parameter space to identify the optimal parameters for use with our selected features. Parameter searches, classifier construction, and PCA were performed in Python 2.7 [66], using the Scikit-learn machine learning toolkit [67]. Plots were made with Matplotlib 1.5.1 [68].

## Additional files

Additional file 1: Predictions and curated annotations of kinase PDB files. This is three column CSV file which states the PDB ID and chain (column 1), the predicted annotation (column 2), and the curated annotation (column 3). (CSV 114 kb)
Additional file 2: Machine learning dataset. This file contains the measured Φ, Ψ, χ1, and pseudo-dihedral angles used in the analysis. (ZIP 15492 kb)

## Abbreviations

ATP: Adenosine tri-phosphate
AUC: Area under curve
CMGC: CDK, MAPK, GSK3 and CLK containing group
PCA: Principal component analysis
PDB: Protein data bank
ProKinO: Protein kinase ontology
PTK: Protein tyrosine kinase
QSAR: Quantitative structure-activity relationship
ROC: Receiver operating characteristic
RSMD: Root mean squared deviation
R-spine: Regulatory spine
STK: Serine/threonine kinase
vdW: Van der Waals
